# Characterization of age-related dolichol increases in the mouse retina

**DOI:** 10.21203/rs.3.rs-6498277/v1

**Published:** 2025-05-16

**Authors:** Oliver Y. Guan, Yiwen Li, Ziqiang Guan, Rong Wen

**Affiliations:** Durham Academy; Bascom Palmer Eye Institute, the University of Miami Miller School of Medicine; Duke University School of Medicine; Bascom Palmer Eye Institute, the University of Miami Miller School of Medicine

**Keywords:** Dolichol, Retina, Mouse, Chain-length profile, Age-related, Postnatal development

## Abstract

We characterized the age-related increases in dolichol levels in the mouse retina using liquid chromatography–mass spectrometry (LC–MS). The four major dolichol species, dolichol-17 (Dol-17), Dol-18, Dol-19, and Dol-20—all increased drastically with age. The largest increase was in Dol-18 levels, which rose by a factor of 100 from postnatal day 5 (PD 5) to PD 600. These increases occurred in two distinct phases: a linear increase in Phase I (PD 5 to PD 30), and a nonlinear increase in Phase II (PD 30 to PD 600). There was also an age-dependent shift in the dolichol chain length profile toward the shorter chain lengths. Dol-19 was the dominant species from PD 5 to PD 15, but Dol-18 became dominant after PD 20. Age-related changes in cholesterol and coenzyme Q9 (CoQ9) were much smaller than those in dolichol but followed the same biphasic pattern. The increase in dolichol levels may influence the physical properties of cell membranes, act as an ultraviolet (UV) filter for retinal cells, and serve as a biomarker of retinal aging.

## Introduction

Dolichol is a homologous series of α-saturated polyisoprenoid alcohols containing 14–24 isoprene units and is ubiquitously present in eukaryotic cells ^1,2^. In mammalian cells, dolichol typically contains 16–23 isoprene units ^3^ and is present in all tissues and most organelle membranes ^2^. Despite the widespread distribution across cell types, the biological and physiological functions of free dolichol are not well understood. In contrast, the function of dolichol phosphate—the phosphorylated derivative of dolichol—is well established as the essential lipid carrier for *N*-linked protein glycosylation ^4–9^.

The levels of dolichol vary substantially across different organs and tissues. In humans, the highest concentrations of free dolichol have been reported in the adrenal gland, pancreas, pituitary gland, testis, and thyroid gland, whereas the lowest amounts were found in the colon, prostate, and placenta ^10^. The range of dolichol levels is so broad that in the pituitary gland, the concentration is more than 100 times higher than that in the placenta ^10^. Such distribution patterns may reflect the specific structural or physiological roles of dolichol in different organs.

The unique importance of dolichol in the retina was demonstrated by causative mutations in the *DHDDS* gene identified in patients with inherited retinal degeneration ^11–14^. The *DHDDS* gene encodes dehydrodolichol diphosphate synthase (DHDDS), a key enzyme in dolichol biosynthesis that catalyzes the *cis*-prenyl chain elongation of dolichol. Patients with these mutations exhibit abnormal blood and urinary dolichol chain length profiles ^12^. Remarkably, these individuals show no other clinical manifestations besides retinal degeneration, suggesting a specific role for dolichol in retinal cells.

The present study focuses on characterizing the levels of major dolichol species in the retinas of mice across an age range from postnatal day 5 (PD 5) to PD 600 using liquid chromatography–mass spectrometry (LC–MS) ^12,15,16^. Our results showed that levels of all four major dolichol species—dolichol-17 (Dol-17), Dol-18, Dol-19, and Dol-20—increase drastically with age in a biphasic pattern: a linear increase in Phase I (PD 5 to PD 30) and a nonlinear increase in Phase II (PD 30 to PD 600). Additionally, an age-related shift in the dolichol chain-length profile was observed. These findings represent an important step toward a better understanding of dolichol’s role in the retina.

## Results

### Increase in dolichol levels with age in the retina

**Figure 1:**
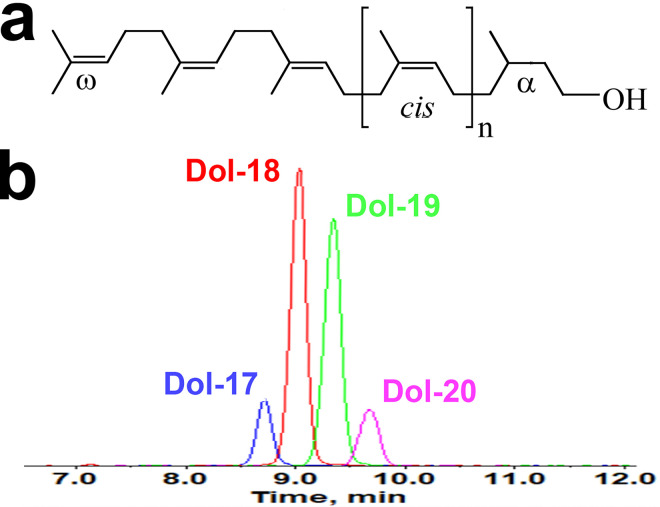
Dolichol profile in the mouse retina. Dolichols are long-chain polyisoprenoid alcohols with a saturated α-isoprene unit, a number (n) of *cis*-units, two *trans*-units at the ω-end and an ω-isoprene unit (**a**). The typical dolichol profile of the retina of a PD30 mouse includes four major dolichol species, Dol-17, Dol-18, Dol-19, and Dol-20—shown by the LC–MRM chromatograms (**b**).

**Figure 2:**
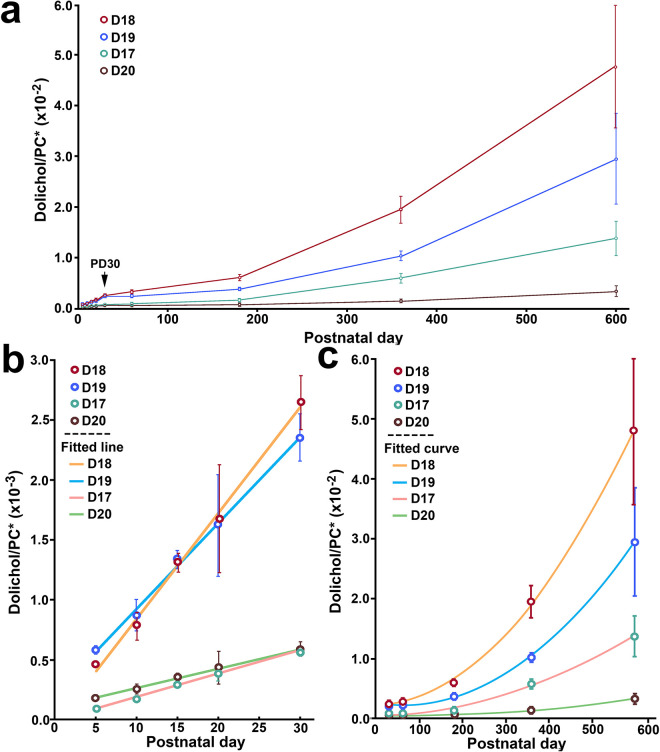
Age-related increase in dolichol in the mouse retina. All four major dolichol species (Dol-17, Dol-18, Dol-19, and Dol-20) increase significantly with age in two phases: Phase I (PD 5 to PD 30) and Phase II (PD 30 to PD 600) (**a**) with PD30 as the transition point (arrow in **a**). In Phase I, increases are linear, and data for each dolichol species are fitted with a straight line (Equation 1) (**b**). In Phase II, increases are nonlinear, with Dol-18 and Dol-19 rising faster than Dol-17 and Dol-20 (**c**). The increase in each species in Phase II are fitted with the quadratic function (Equation 2) (**c**). PC* denotes the average of the two most abundant retinal PC species used for normalization: PC (16:0/16:0) and PC (16:0/18:1).

Dolichol is a series of long-chain polyisoprenoid alcohols containing a saturated α-isoprene unit, as shown in Fig. 1a. A typical mouse retinal dolichol profile includes four major dolichol species: Dol-17, Dol-18, Dol-19, and Dol-20 (Fig. 1b). As shown in Fig. 2a, all four major retinal dolichol species increased with age. The two dominant species, Dol-18 and Dol-19, increase faster than Dol-17 and Dol-20.

To compare dolichol levels at different ages, the level of each dolichol species measured by LC – MS in each sample was normalized to the level of the major membrane phospholipid phosphatidylcholine (PC) in the same sample. The normalized data reveal that all four dolichol species increase significantly with age in a biphasic pattern (Fig. 2a). In the first phase (Phase I, from PD 5 to PD 30), the increases appear linear, whereas in the second phase (Phase II, from PD 30 to PD 600), the increases are nonlinear. The transition point between the two phases is at PD30, indicated by an arrow in Fig. 2a.

We further analyzed increases in each dolichol species in Phase I and Phase II separately. In Phase I (PD 5 to PD 30), the increases in dolichol levels were fitted with a linear function:

Eq. 1 f(x)=ax+b

where x represents the age of the animal (in postnatal day) and f(x) represents the dolichol level (Fig. 2b). The slopes for Dol-18 and Dol-19 are comparable, as are those for Dol-17 and Dol-20 (Fig. 2b).

In contrast, during Phase II (PD 30 to PD 600), the levels of Dol-18 and Dol-19 increase faster than those of Dol-17 and Dol-20 (Fig. 2c). The data for each dolichol species were fitted with the quadratic function:

Eq. 2 $$f(x)=ax^{2}+bx+c$$

where x and f(x) are defined above. The parameters of the fitted equations (Eqs. 1 and 2) for each dolichol species are listed in Supplementary Table 1 online.

### Age-related shift in dolichol chain length profile in the retina

**Figure 3:**
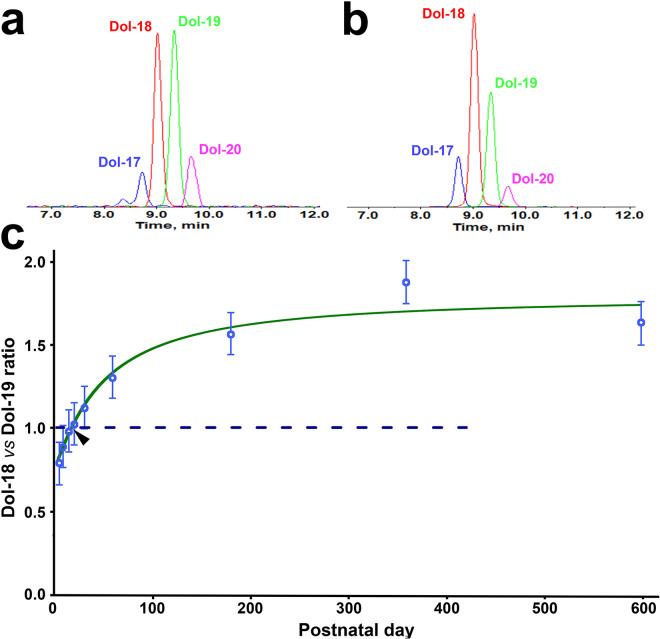
Age-related shift in dolichol chain length profile. In LC-MRM chromatograms of a PD10 retina, the Dol-18 peak area is smaller than that of Dol-19 (**a**), whereas in a PD180 retina, the Dol-18 peak area exceeds that of Dol-19 (**b**; panels **a** and **b** are at different vertical scales). The Dol-18 to Dol-19 ratio, plotted across ages in panel **c,** is fitted with a four-parameter logistic curve (Equation 3, green curve). The fitted curve shows that the ratio increases steadily from PD5 to PD60 and reaches a plateau by PD180. At a point between PD 15 and PD20 (arrow in **c**), the ratio reaches 1. The dashed line in panel **c** indicates where the ration equals 1.

Each dolichol species increases at a different rate (Fig. 2), resulting in a shift in dolichol chain-length distribution. For example, Dol-19 is the most abundant dolichol species at early ages (Fig. 2b), but Dol-18 rises at a faster rate and becomes the most abundant species later (Fig. 2b, c). Similarly, Dol-17 is the least abundant species at early ages (Fig. 2b), but Dol-20 becomes the least abundant species later (Fig. 2b, c). As shown in Fig. 3, in the LC-MRM chromatograms of a PD10 retina, the Dol-19 peak area is greater than that of Dol-18 (Fig. 3a). However, in the LC-MRM chromatograms of a PD180 retina, the Dol-18 peak area exceeds that of the Dol-19 (Fig. 3b). Thus, the dolichol chain length profile shifts toward the shorter chain lengths with age. To quantify this shift, we calculated the ratio of Dol-18 to Dol-19, the two dominant dolichol species. The Dol-18 level is lower than that of Dol-19 before PD 15 (Fig. 3c, data below the dash line) but exceeds it after PD 20 (Fig. 3c, data above the dash line). The Dol-18 to Dol-19 ratio is 1 at a point between PD 15 and PD 20 (Fig. 3c).

The age-related increase in Dol-18 to Dol-19 ratio was fitted with a four-parameter logistic function:

Eq. 3 $$f(x)=d+(a-d)/(1+{\left(\frac{x}{c}\right)}^{b})$$

where x represents the age of the animal (in postnatal day) and f(x) denotes the value of Dol-18 to Dol-19 ratio (Fig. 3c green curve). The fitted curve indicates that the ratio’s increase at a nearly constant rate curve from PD5 to PD 60, and reaching a plateau by PD180 (Fig. 3c). The parameters of the fitted function (Eq. 3) are listed in Supplementary Table 2 online.

### Levels of cholesterol and coenzyme Q9 in the mouse retina

**Figure 4:**
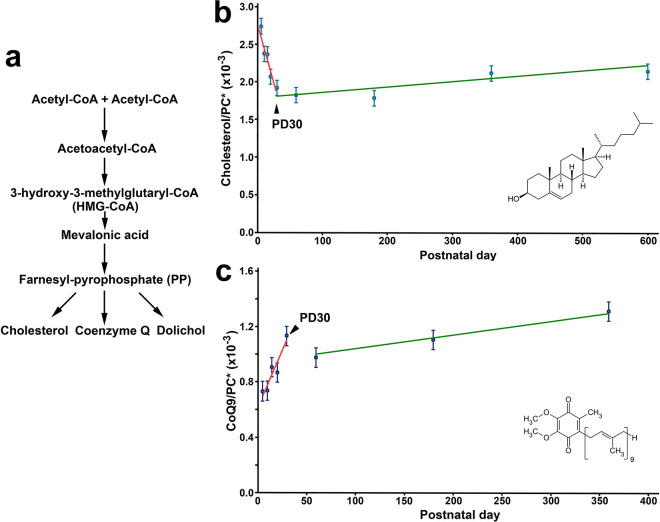
Age-related changes in cholesterol and CoQ9 in the mouse retina. The mevalonate pathway, shared by dolichol, cholesterol, and CoQ9, is shown in panel **a**. Cholesterol levels change in two phases, a linear decrease in Phase I (PD 5 to PD 30) and a gradual linear increase in Phase II (PD 30 to PD 600) (**b**). The decrease in Phase I and increase in Phase II are fitted with linear functions (orange and green lines in **b**, respectively). CoQ9 levels also change in two phases, a linear increase in Phase I (PD 5 to PD 30), followed by a decrease from PD 30 to PD 60 and gradual increase to PD 360 in Phase II (**c**). The straight lines represent fitted trends for the increase in Phase I (orange line in **c**) and the increase in Phase II (green lines in **c**). The transition point at PD30 is indicated by arrows in **b** and **c**. Insets in **b** and **c**show chemical structures of cholesterol and CoQ9, respectively.

We measured the levels of cholesterol and coenzyme Q9 (CoQ9, the most abundant ubiquinone species in mice) in each sample for comparison with dolichol. Cholesterol and CoQ9 share the same upstream mevalonate pathway with dolichol (Fig. 4a). As for the levels of dolichol, the level of cholesterol or CoQ9 was also normalized to the level of PC in the same sample.

Age-related changes in cholesterol level also occur in two phases (Fig. 4b). In Phase I (PD 5 to PD 30), cholesterol levels decrease linearly to 70% of the PD 5 level (Fig. 4b). In Phase II, (PD 30 to PD 600) the levels gradually increase to 78% of the PD5 level (Fig. 4b). These changes were fitted with linear function f(x)=ax+b for the decrease in Phase I and linear function f(x)=a′x+b′ for the increase in Phase II (Fig. 4b, orange line and green line, respectively).

Similarly, CoQ9 levels exhibit biphasic age-related changes (Fig. 4c). In Phase I (PD 5 to PD 30), CoQ9 levels increase linearly to 154% of the PD 5 level (Fig. 4c). In Phase II (PD 30 to PD 360), CoQ9 levels drops to 133% of the PD 5 level by PD 60 and gradually increases to 178% of the PD 5 level at PD 360 (Fig. 4c). These changes were fitted with linear function f(x)=ax+b for the increase in Phase I and linear function f(x)=a′x+b′ for the increase in Phase II between PD 60 to PD 360 (Fig. 4c, orange line and green line, respectively)

The parameters in the fitting lines shown in Fig. 4b and 4c are listed in in Supplementary Tables 3 and 4, respectively, online.

## Discussion

We have demonstrated a dynamic, age-related increase in all four major dolichol species in the mouse retina, including Dol-17, Dol-18, Dol-19, and Dol-20. The largest increase occurs in Dol-18, which increases 100-fold from PD 5 to PD 600. These increases occur in two phases: Phase I (PD5 to PD30), and Phase II (PD30 to PD600). In Phase I, dolichol levels increase linearly (Fig. 2a and 2b), whereas in Phase II, the increases are nonlinear, following a quadratic curve (Fig. 2a and 2c). The transition point between the two phases is PD30. Notably, cholesterol and CoQ9 levels also exhibit biphasic changes with P30 as the transition point (Fig. 4b and 4b).

The mouse retina continues to develop postnatally until PD30, at which point it is considered fully mature ^17^. Thus, our data show distinct dolichol increases during postnatal development (Phase I) and from young adulthood to old age (Phase II) ^18^. Interestingly, an age-related increase in total dolichol in the rat brain has been reported to occur in two phases ^19^. The first phase extends from birth to PD 50, and the second phase occurs from PD 60/70 to PD 330 ^19^.

Alongside the increase in dolichol levels, we observed an age-related shift in the dolichol chain length profile (Fig. 3). Dolichol chain-length profile is species-specific ^3,20^ and is regulated by the length-determination mechanism. Manipulation of the regulatory domains in *cis*-prenyltransferase has been shown to alter the prenyl chain lengths ^21^. The shift toward shorter chain lengths observed in our study suggests a modification of the dolichol length-determination mechanism in the retina during development.

The dolichol length profile is particularly relevant to the retina. In patients with DHDDS mutations, which cause inherited retinal degeneration, these mutations also lead to shortened dolichol chain length in serum and urine ^12^. Although the mutations carried by the patients were initially believed to cause a defect in *N*-linked protein glycosylation ^11,14^, a later study found that affected patients exhibited a normal patten of blood transferrin isoelectric focusing, a test widely used to screen for congenital disorders of glycosylation ^12^. Thus, a defect in protein glycosylation is unlikely the primary cause of retinal degeneration in these patients. Interestingly, patients with neurodevelopmental and neurodegenerative disorders caused by other DHDDS mutations exhibit a normal dolichol length profile and no retinal degeneration ^22^. Further investigation into the mechanisms linking altered dolichol chain length profile to DHDDS-associated retinal degeneration could provide valuable insights.

The mechanism underlying the aging-related increase in dolichol in the retina remains unknown. In the adult mouse brain, dolichol synthesis has been reported to be low ^23^. It is likely that dolichol synthesis in adult mouse retina is similarly low. Therefore, it is unlikely that the age-related increase in retinal dolichol results from an increase in dolichol synthesis rate. On the other hand, dolichol degradation is slow ^24^, with no enzymatic degradation of dolichol has been demonstrated ^16,25^. Additionally, dolichol is chemically and biochemically stable ^16^. Therefore, the age-related increase likely results from accumulation over time rather than increased synthesis.

The substantial increase of dolichol raises a question of how high dolichol levels affect retinal cell on function and biology. Despite its discovery over six decades ago ^26,27^, the biological role of free dolichol remains poorly understood ^3,28^. Studies on model membranes suggest that dolichol increases membrane fluidity and facilitates vesicle fusion ^29,30^. Experimental evidence also indicates dolichol protects of membrane lipids from oxidative damage caused by free-radicals ^31^. Additionally, dolichol absorbs ultraviolet (UV) irradiation, and its UV-absorbing capacity increases significantly with UV exposure ^32^. Thus, elevated dolichol levels may enhance the UV-blocking capacity of retinal cells.

Age-related increases in dolichol have been reported in brain, liver and other tissues in mice, rats, and humans ^2,19,33–37^. It has been suggested that age-related tissue accumulation of dolichol may serve as a biomarker of aging ^34,38^. Similarly, dolichol accumulation in the retina could function as a biomarker of retinal aging, providing a tool for research into retinal aging and pathogenesis of age-related retinal diseases.

## Methods

### Animals

Experimental procedures were approved by the Institutional Animal Care and Use Committee (IACUC) of the University of Miami, Miller School of Medicine. All methods were carried out in accordance with relevant guidelines and regulations, including adherence to the ARRIVE (Animal Research: Reporting of In Vivo Experiments) guidelines and the ARVO (Association for Research in Vision and Ophthalmology) Statement for the Use of Animals in Ophthalmic and Vision Research. Wild-type C57BL/6 mice ranging in ages from PD5 to PD600 were purchased from Jackson Labs (Bar Harbor, ME) for retinal lipidomic analysis.

### Retinal lipid extraction

To extract retinal lipids, animals were euthanized by CO_2_ overdose and their eyes were collected. The anterior segment of each eye was removed, and the retina was carefully detached and collected. Total lipids were extracted from each retinal sample using a modified Bligh and Dyer method ^39^ as described previously ^12,40^. Briefly, each sample was homogenized with 100 μL of H_2_O in a Bullet Blender (Next Advance, Troy, NY). The homogenate was then mixed with 100 μL of methanol in the Bullet Blender, followed by the addition of 100 μL of chloroform (CHCl_3_) and mixing again. The lipid-containing chloroform was separated from the rest of the mixture by centrifugation and transferred to a collection tube. Lipids in the chloroform from each retina were dried in a SpeedVac (Savant Instruments, Holbrook, NY), flushed with argon, and stored at − 20°C in the dark until use.

### LC-MS analysis

Retinal lipids were analyzed by LC-MS ^12,41^ performed in multiple reaction monitoring (MRM) mode using a Shimadzu LC system (comprising a solvent degasser, two LC-10A pumps and a SCL-10A system controller) coupled to a 4000 Q-Trap hybrid triple quadrupole linear ion-trap mass spectrometer equipped with a Turbo V ion source (AB-Sciex, Foster City, CA). LC was operated at a flow rate of 200 μl/min with a linear gradient as follows: 100% of mobile phase A was held isocratically for 2 min and then linearly increased to 100% mobile phase B over 14 min and held at 100% B for 4 min. Mobile phase A consisted of methanol/acetonitrile/aqueous 1 mM ammonium acetate (60/20/20, v/v/v). Mobile phase B consisted of 100% ethanol containing 1 mM ammonium acetate. A Zorbax SB-C8 reversed-phase column (5 μm, 2.1×50 mm) was obtained from Agilent (Agilent Technologies, Santa Clara, CA).

The major retinal dolichol species, Dol-17, Dol-18, Dol-19, and Dol-20 (the number represents the numbers of isoprene unit), were measured by MRM performed in the negative ion mode with MS settings as follows: Curtain Gas (CUR) = 20 psi (pressure), Gas-1 (GS1) = 20 psi, Gas-2 (GS2) = 30 psi, Ion Spray voltage (IS) = − 4500 V, Source Temperature (TEM) = 350°C, Interface Heater = ON, De-clustering Potential (DP) = − 40V, Entrance Potential (EP) = − 10V and CXP = − 5V. The voltage used for collision-induced dissociation was − 40V (laboratory frame of energy). Nitrogen was used as a gas collision. The MRM pairs for Dol-17, Dol-18, Dol-19, and Dol-20 were 1236.2/59, 1304.2/59, 1372.2/59, and 1440.3/59, respectively. In these MRM pairs, the precursor ions are the [M + acetate]^−^ adduct ions, and the product ions are the acetate ions (*m/z* 59). Cholesterol ^42^ and coenzyme Q9 ^15^ were measured in the positive ion mode. For correcting experimental variations and relative quantitation, dolichols were normalized with phosphatidylcholine (PC), the most abundant membrane phospholipid in the retina, in the same sample. The MRM pairs (precursor/product) for the detection of dolichol, CoQ9, cholesterol and PC are listed in Table 1.

### Data Analysis

Data modeling and analyses were performed using software MATLAB (The MathWorks, Inc. Natick, MA). All data are presented as mean ± standard deviation (SD) (n = 3).

## Figures and Tables

**Table 1:** The MRM pairs

	Ion Mode	Precursor ion	Product ion
Dol	Negative	[M + Ac]^−^	59
PC	Negative	[M + Ac]^−^	[M-14]^−^
CoQ9	Positive	[M + NH_4_]^+^	197
Cholesterol	Positive	[M + H]^+^	369

## Data Availability

Datasets from this study are available from the corresponding authors upon reasonable request.
